# Etiological spectrum and diagnostic features of lymphadenopathy in People Living with HIV in French Guiana: A 17-years multicenter retrospective case series

**DOI:** 10.1371/journal.pntd.0013558

**Published:** 2025-09-22

**Authors:** Morgane Bourne-Watrin, Ugo Françoise, Sophie Alexandra Baron, Antoine A. Adenis, Dufens Pierre Louis, Loïc Epelboin, Mathieu Nacher, Félix Djossou, Kinan Drak Alsibai, Pierre Couppié

**Affiliations:** 1 Service de Dermatologie-Vénérologie, Centre Hospitalier de Cayenne, Cayenne, Guyane française; 2 Centre d’Investigation Clinique Antilles Guyane, Inserm CIC 1424, Centre Hospitalier de Cayenne, Cayenne, Guyane française; 3 Coordination de la lutte contre les infections sexuellement transmissibles et le virus de l’immunodéficience humaine de la Guyane, Centre Hospitalier de Cayenne, Cayenne, Guyane française; 4 Institut Santé des Populations en Amazonie, Centre Hospitalier de Cayenne, Cayenne, Guyane française; 5 Institut Pasteur de la Guyane, Cayenne, Guyane française; 6 Service de Médecine A, Centre Hospitalier de Cayenne, Cayenne, Guyane française; 7 Unité des Maladies Infectieuses et Tropicales, Centre Hospitalier de Cayenne, Cayenne, Guyane française; 8 TBIP, Université de Guyane, Université de Lille, CNRS, Inserm, Institut Pasteur de Lille, U1019-UMR9017-CIIL Centre d’Infection et d’Immunité de Lille, Cayenne, Guyane française; 9 Service d’Anatomie et Cytologie Pathologique et Centre de Ressources Biologiques (CRB Amazonie), Centre Hospitalier de Cayenne, Cayenne, Guyane française; 10 Registres des Cancers de Guyane (RCan Guyane), Département de Recherche et d’Innovation en Santé Publique (DRISP), Centre Hospitalier de Cayenne, Cayenne, Guyane française; Albert Einstein College of Medicine, UNITED STATES OF AMERICA

## Abstract

**Introduction:**

Lymph node enlargement can be present at any stage of the HIV infection, reactive to HIV itself, another infection, or a malignant source. The aim of our study was to describe the causes of lymphadenopathy in the people living with HIV (PLHIV) of French Guiana, a French territory in the Amazon region.

**Methods:**

A retrospective multicenter case series was conducted between January 2005 and December 2021. Inclusion population consisted of PLHIV who underwent a fine needle aspiration or a biopsy of a lymph node that was analyzed at the Department of Pathology of Cayenne hospital.

**Results:**

We included 152 adults, with a median age of 43 [35–51] years and median CD4 count of 185/mm^3^ [60–344]. The main causes of lymphadenopathy were: histoplasmosis (25%, CI95%: 18–33), followed by tuberculosis (24%, CI95%: 18–32), HIV-reactive lymphadenitis (21%, CI95%: 15–29) and lymphoproliferative disorder (11%, CI95%: 7–18). Multiple causes were present in 6% of cases. Opportunistic infections represented 53% (CI95%: 44–61) of cases. The main characteristics associated with opportunistic disease (infectious or neoplastic) were lymph node > 5 cm, CD4 count < 200/mm^3^, hepatomegaly or splenomegaly and the presence of extra-ganglionic symptoms.

**Conclusion:**

For the last 17 years, opportunistic infections represented 53% of the causes of lymphadenopathy among PLHIV. Histoplasmosis, already recognized as the first AIDS-defining condition and first cause of AIDS-related deaths in French Guiana, is also the first cause of lymphadenopathy in PLHIV, ahead of tuberculosis and reactive lymphadenopathy. The CD4 count and the size of the lymph nodes appear to be the most important factors in the diagnostic process and should lead to a quick lymph node analysis in these patients.

## Author summary

Lymph node enlargement is common during the course of the HIV infection, reactive to HIV itself, or due to an opportunistic infectious or neoplastic cause that can put the life of patients at risk. Thus, it is important to distinguish reactive or normal lymph nodes from opportunistic causes needing a biopsy to quickly introduce a specific treatment if necessary. We studied the causes of lymphadenopathy requiring a biopsy or a fine needle aspiration in people living with HIV over 17 years in French Guiana. In this territory in the Amazon region with major socioeconomic inequalities, a universal health system and an Amazonian pathogen ecosystem, we hypothesized that the main etiologies may differ from those of temperate regions, with a higher proportion of opportunistic infections. In our population mainly composed of immunocompromised patients with < 200 CD4/mm3, in a region with high histoplasmosis and tuberculosis endemicity, opportunistic infections and malignancy were more than twice as common as benign causes. The CD4 count, size of the lymph nodes and presence of associated respiratory signs or fever appear to be the most important factors in the diagnostic process and should lead to a quick lymph node analysis in these fragile patients.

## Introduction

Lymph nodes are secondary lymphoid organs that play a major role in adaptive immunity. Since the onset of the human immunodeficiency virus (HIV) pandemic, lymphadenopathy has been considered a clinical manifestation of the disease [1], with HIV initially referred to as the “lymphadenopathy-associated virus”. In people living with HIV (PLHIV), enlarged lymph nodes are most often reactive adenitis, or secondary to Hodgkin’s and non-Hodgkin’s lymphoma and tuberculosis [2]. HIV-reactive lymphadenopathy is defined as a palpable lymphadenopathy > 1 cm involving at least two non-continuous lymphatic areas, lasting for more than 3 months, after the exclusion of another concomitant disease [3]. This reactive state must be differentiated from all other infectious and non-infectious etiologies which require specific treatment.

French Guiana is a French overseas territory located in South America, bordering Suriname and Brazil. The population is multiethnic, mainly composed of Amerindians, Creoles, Bushinenges (African-American Maroons), French from mainland France, Hmong (originated from Laos) and Chinese. In addition, people migrating from the neighboring territories, mainly Haiti, Brazil, Suriname and Guyana represent a substantial part of the population [4]. These migratory histories explain the singular epidemiology of HIV infection in French Guiana and notably the varying difficulties of care between these populations [5]. A high level of health care, notably HIV infection care and treatment according to the standards of high-income countries, is available in the three main hospitals (Cayenne, Saint-Laurent-du-Maroni and Kourou) and in the Delocalized Centers for Prevention and Care (DCPC) in the more remote towns. French Guiana has a remarkable human diversity, with major socioeconomic inequalities, a universal health system and an Amazonian pathogen ecosystem. In this context, we hypothesized that the main etiologies of lymphadenopathy may differ from those of temperate regions, with a higher proportion of opportunistic infections requiring specific management.

Hence, the main objective of the present study was to describe the etiological spectrum of lymphadenopathy among PLHIV under follow-up in French Guiana. The secondary objective was to identify clinical and paraclinical features associated with AIDS (Acquired Immunodeficiency Syndrome)-defining opportunistic conditions when compared to HIV-reactive or benign etiologies.

## Methods

### Ethics statement

All participants of the present study were included in the DMI-2/ DOMEVIH database, administered by the local Regional Coordination for the fight against HIV (COREVIH Guyane). This database is part of the French Hospital Database on HIV, a French national cohort of PLHIV since 1992, that has received approval by the French regulatory authority, the Commission Nationale Informatique et Libertés (CNIL), on November 27, 1991. The main objective of this hospital cohort is to study the evolution, morbidity and mortality of PLHIV in French Guiana [6]. Socio-demographic, clinical, paraclinical and therapeutic data have been retrospectively collected since January 1^st^, 1992 for all PLHIV under follow-up in public hospitals of French Guiana. Oral and written information was provided and all participants signed an informed consent form for the cohort study and related studies together with data completion of missing data overtime and publication of results.

### Study design and study population

We carried out a retrospective multicenter study of PLHIV who underwent lymph-node sampling between January 1^st^ 2005 and December 31^st^ 2021 and abstracted clinical, laboratory and imaging data at the time of the presentation. The source population was all known PLHIV in whom a cytological or histological examination (CHE) by fine needle aspiration (FNA) or biopsy of a lymphadenopathy was addressed to the Department of Pathology of Cayenne Hospital. The pathology department in Cayenne is the reference center in French Guiana and receives samples from the three public hospitals and from the DCPC.

### Inclusion criteria

Inclusion criteria were age > 18 years and confirmed HIV infection (2 positive serologies and Western blot). Patients with only non-contributive samples (muscle, fat, necrosis, structure other than a lymph node, or any tissues too damaged to be analyzed) were excluded.

### Case definition

Two samples collected for the same medical complaint/event were considered as a single event.

Lymph node areas were gathered according to the FLIPI nodal map [7]. Sub-maxillary, parotid, pre-thyroidal, pre-tracheal, under-mental and supraclavicular lymph nodes were considered as cervical sites; Iliac and latero-aortic lymph nodes were gathered in para-aortic site. Gastric, peri-vesicular, colic, and pelvic lymph nodes were considered as abdominal lymphadenopathy; spinal lymph nodes were separated when their anatomic level was not known.

HIV-reactive lymphadenitis included: hyperplastic adenitis, follicular adenitis with or without histiocytosis, lymphocytic depletion attributed to HIV and angioimmunoblastic lymphadenopathy when attributed to HIV by the pathologist. If a patient presented a reactive adenitis with a disseminated infection such as tuberculosis but without evidence of tuberculosis in the lymph node, the lymphadenopathy was considered as non-specific adenitis (and not as HIV-reactive).

For human herpesvirus 8 (HHV8) associated cancers, Castleman’s disease was considered as a lymphoproliferative disorder (LPD); Kaposi sarcoma was not included in the metastases nor LPD group, since it presents a complex and still not totally understood physiopathology with an invasion linked to neo-angiogenesis and not to lymphoid diffusion [8,9].

For infections, microbiological diagnosis was made in histology based on the presence of Acid-fast bacilli with the Ziehl-Neelsen stain or yeast with the Gomori-Grocott stain or using specific tuberculosis or histoplasmosis PCR. Architectural transformations (granuloma, necrosis…) were not considered as specific of a pathology.

Mycological and mycobacteriological direct examination or culture (stored for 6 and 9 weeks respectively) could also be positive.

Diagnosis was only confirmed if the pathogen was found in the lymph node. A positive result in another organ was not sufficient to confirm the diagnosis. Positive histoplasmosis or cryptococcal antigen alone was not used as diagnosis criteria.

### Etiological groups

Five etiological groups were initially defined, with as main causes: HIV-reactive, tuberculosis, histoplasmosis, lymphoma and metastasis.

In order to answer the secondary objective, which was to identify clinical and paraclinical features associated with AIDS-defining opportunistic conditions, we separated the patients into two groups: opportunistic and benign/reactive causes. AIDS-defining opportunistic conditions included: histoplasmosis, tuberculosis and other mycobacterial infections, cryptococcosis (defined as opportunistic infections), Kaposi sarcoma and LPD. Patients with normal lymph nodes and HIV-reactive lymphadenitis were included in the benign/reactive group. We excluded patients with solid cancer metastases from the comparative analysis since their lymphadenopathy had been sampled as part of an extension work-up for a previously identified cancer but not for diagnostic purposes. Patients with reactive adenitis not attributed to HIV or any associate opportunistic disease diagnosed outside of the lymph node were also excluded from this analysis.

### Data collection procedure

We screened for all lymph node samples in the Diamic (Dedalus, France) pathology department software using the code “SG” over the study period and excluded non-contributive samples. Medical reports were then screened to search for PLHIV within the selected samples. For all eligible patients, data collected from the pathology department included Gram, Periodic-Acid-Shift, Gomori-Grocott and Ziehl-Neelsen staining, and were cross-checked with the ones of the microbiological laboratory.

Data were retrospectively and systematically collected on a standardized form using Epidata software, and stored in an anonymized Excel database. We collected the etiologies of lymphadenopathy and their relevant sociodemographic, clinical and paraclinical associated data from the patients’ medical files within the French Guiana PLHIV cohort database (DMI-2-DOMEVIH, French Hospital Database on HIV, FHDH).

### Data analysis

Quantitative variables were presented with median and 25%-75% interquartile ranges [IQR], categorical variables with proportion and confidence interval when relevant. Association between AIDS-defining opportunistic conditions and explanatory variables were tested with Wilcoxon, **χ**^**2**^ or Fisher exact test according to the quality of the variables. A multivariate explaining model was developed using Firth’s penalized logistic regression. Variables were first screened in bivariate analyses with a significance threshold of p < 0.20. Variables were then retained if they represented a clinically relevant difference. Among highly correlated variables (Pearson’s correlation coefficient > 0.7), those considered less clinically relevant or with more missing data were excluded. The remaining variables were entered into a manual step-down selection process guided by the Akaike Information Criterion. Sex was included as an adjustment variable. Statistical analyses were performed using R studio version 4.3.2.

Patients included before 2016 and presenting with > 500 CD4/mm^3^ were not included in the analysis concerning treatment intake, since it was not strictly recommended to treat them at that time following the French national recommendations [10,11].

## Results

### Baseline characteristics

**Fig 1:**
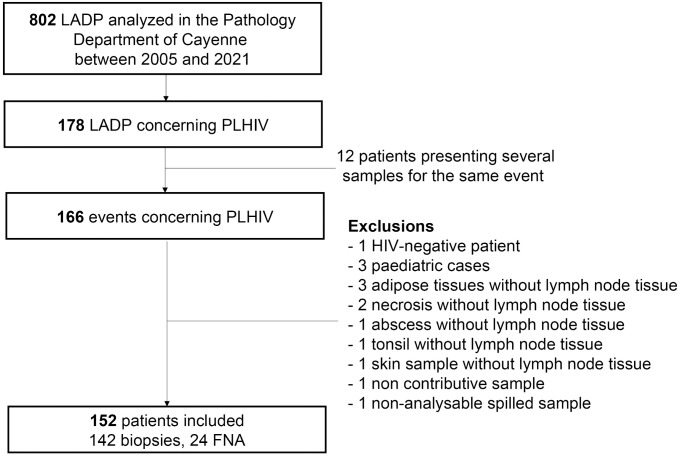
Flow chart 2005-2021. Abbreviations: FNA: Fine Needle Aspiration; LADP: Lymphadenopathy; PLHIV: People Living with the Human Immunodeficiency Virus.

Over the 17 years study period, 152 PLHIV were included, representing 142 biopsies and 24 FNA (Fig 1).

The male to female sex ratio was 1.6 and the median age was 43 [35–51] years (Table 1).

**Table 1:** Baseline data of 152 cases of lymphadenopathy in PLHIV between 2005 and 2021.

	No/Total (%) Median [IQR25%-75%]
**Sex ratio Male/Female**	1.6
**Median age (years)**	43 [35-51]
**Place of birth** (n = 149)	
Haiti	51/149 (34)
French Guiana	27/149 (18)
Brazil	25/149 (17)
Suriname	19/149 (13)
Guyana	17/149 (11)
Other^*^	10/149 (7)
**Transmission mode of HIV** (n = 149)	
Heterosexual	126/149 (85)
Homosexual	2/149 (1)
Bisexual	2/149 (1)
Materno-fetal	1/149 (1)
Unknown	18/149 (12)
**Antiretroviral therapy at diagnosis**^†^ (n = 148)	54/148 (36)
**Median CD4 count (cells/mm3)** (n = 145)	185 [60-344]
**Median HIV viral load (log10 copies/ml)** (n = 140)	4.5 [2.1-5.3]
**Time interval between diagnosis of HIV and actual diagnosis** (n = 148)	
[1-6 months]	46/148 (31)
[6 months-2 years]	25/148 (17)
[2-5 years]	28/148 (19)
[5-10 years]	20/148 (14)
≥ 10 years	29/148 (20)
**Concomitant extra-ganglionic AIDS-defining conditions**^‡^ (n = 149)	28/149 (19)
Esophageal candidiasis	10/149 (7)
Cerebral toxoplasmosis	7/149 (5)
Extra-ganglionic tuberculosis	5/149 (3)
CMV disease	4/149 (3)
Extra-ganglionic histoplasmosis	3/149 (2)
Extra-ganglionic *Mycobacterium avium complex* infection	2/149 (1)
HIV associated nephropathy	2/149 (1)
Recurrent mucocutaneous infections due to HSV	2/149 (1)
Other^§^	4/149 (3)

Abbreviations: AIDS: Acquired Immunodeficiency Syndrome; CMV: Cytomegalovirus;

HSV: Herpes Simplex Virus; IQR: interquartile ranges; No: Number.

n = number of available data; Medians are presented with 25–75% interquartile ranges in brackets.

*Other place of birth: Mainland France (n = 4), Dominican Republic (n = 2), Martinique (n = 1), Guinea-Bissau (n = 1), Venezuela (n = 1), Senegal (n = 1);

†Patients with a newly diagnosed HIV infection and patients included before 2016 and presenting more than 500 CD4/mm3 were not included since it was not strictly recommended to treat them that time following national recommendations;

‡Multiple associated conditions possible. As the wasting syndrome could be secondary to the cause of the lymphadenopathy, we did not consider this syndrome as an associated AIDS-defining condition;

§Other concomitant extra-ganglionic AIDS-defining conditions: *Salmonella* sp. bacteremia (n = 1), *Pneumocystis jirovecii* pneumonia (n = 1), Chronic intestinal cryptosporidiosis (n = 1), Cutaneous Kaposi sarcoma (n = 1).

Patients were mainly born in Haiti (34%), French Guiana (18%), and Brazil (17%). The main transmission mode of HIV was heterosexual (85%). Median CD4 count and HIV viral load were 185/mm3 [60–344] and 4.5 log10 copies/ml [2.1-5.3], respectively. Almost 20% of patients presented concomitant extra-ganglionic AIDS-defining conditions, mainly composed of esophageal candidiasis and cerebral toxoplasmosis. At the time of analysis of lymphadenopathy, the diagnosis of HIV was known for less than 6 months in 31% of cases. Missing data for Bacillus Calmette-Guérin (BCG) vaccination and environmental exposures were too numerous to include these variables.

### Main etiologies of lymphadenopathy

The main lymphadenopathy etiologies were histoplasmosis (38/152, 25%, CI95%: 18–33), followed by tuberculosis (37/152, 24%, CI95%: 18–32) and HIV-reactive lymphadenitis (32/152, 21%, CI95%: 15–29) (Table 2). Lymphoproliferative disorder represented 11% (17/152, CI95%: 8–17) of cases, mainly Hodgkin lymphoma (7/152, 5%). Opportunistic infections represented 53% (80/152, CI95%: 44–61) of cases. Seven lymph nodes analyzed were normal. Multiple associated causes were found in 9 patients (6%), mainly composed of histoplasmosis and mycobacterial infection and/or LPD. Six patients (4%) presented an associated Immune Reconstitution Inflammatory Syndrome (IRIS), defined as an excessive inflammatory response to a pathogen, attributed to rapid restoration of the immune system following introduction of antiretroviral therapy [12].

**Table 2:** Main etiologies of lymphadenopathy in PLHIV, 2005-2021, French Guiana.

	No/Total (%)
**Histoplasmosis**	**38/152 (25)**
Associated^*^ LPD	4/38 (11)
Associated^*^ tuberculosis	3/38 (8)
Associated^*^ non-tuberculosis mycobacterial infection	2/38 (5)
**Tuberculosis**	**37/152 (24)**
Associated^*^ histoplasmosis	3/37 (8)
Associated^*^ LPD	1/37 (6)
**HIV-reactive lymphadenitis**	**32/152 (21)**
**Lymphoproliferative disorder**	**17/152 (11)**
Hodgkin lymphoma (scleronodular)	7/152 (5)
Associated^*^ histoplasmosis and tuberculosis	1/7 (14)
T-cell lymphoma	4/152 (3)
Angio-immunoblastic T-cell lymphoma (EBV+)	2/152 (1)
Peripheral T-cell lymphoma Not Otherwise Specified	1/152 (1)
Associated^*^ histoplasmosis	1/1 (100)
HTLV-associated T-cell lymphoma	1/152 (1)
Associated^*^ histoplasmosis	1/1 (100)
B-cell lymphoma	4/152 (3)
Diffuse large B-cell lymphoma	2/152 (1)
Burkitt’s lymphoma	1/152 (1)
Plasmablastic lymphoma (EBV+)	1/152 (1)
Castleman disease	2/152 (1)
**Solid tumor metastasis (primitive site)**	**9/152 (6)**
Breast	2/152 (2)
Digestive tract ^†^	3/152 (2)
Pelvis^‡^	2/152 (4)
Larynx	1/152 (1)
Melanoma	1/152 (1)
**Kaposi sarcoma**	**2/152 (1)**
Associated^*^ tuberculosis	1/2 (50)
**Non-tuberculous mycobacteria**	**8/152 (5)**
MAC	6/152 (4)
Associated^*^ histoplasmosis	1/6 (17)
Non-identified mycobacteria	2/152 (1)
Associated^*^ histoplasmosis	1/2 (50)
**Cryptococcosis**	**2/152 (1)**
**Other inflammation**	**11/152 (7)**
Non-specific adenitis	9/152 (6)
Necrotic adenitis	1/152 (1)
Epithelioid and giant cell granuloma without necrosis	1/152 (1)
**Normal**	**7/152 (5)**
**Multiple associated causes**	**9/152 (6)**
**Associated IRIS** ^§^	**6/152 (4)**

Abbreviations: EBV: Epstein–Barr virus; HTLV: Human T-Lymphotropic Virus; IRIS: Immune Reconstitution Inflammatory Syndrome; LPD: Lymphoproliferative Disorder; MAC: *Mycobaterium Avium* Complex infection.

*Associated diagnosis concerning lymph node’s involvement;

†1 independent cell gastric carcinoma, 2 Lieberkuhnian carcinoma of the colon;

‡1 epidermoid endometrial carcinoma, 1 epidermoid carcinoma of the penis;

§3 tuberculosis, 1 non-tuberculous mycobacteria and histoplasmosis, 1 MAC, 1 histoplasmosis.

### Microbiological and pathological diagnosis

In case of histoplasmosis or tuberculosis, diagnosis was mainly made by culture (75% (21/28) and 65% (15/23)) or CHE (77% (23/30) and 59% (17/29)) (Table 3), contrary to non-tuberculous-mycobacteria where positive direct examination was the most conclusive exam in 80% (4/5). Cryptococcosis could only be diagnosed by CHE.

**Table 3:** Diagnostic tools in case of infectious disease (including patients presenting several concomitant causes of lymphadenopathy) (No/Total (%)).

Lymph node analysis	Histoplasmosis (n = 38)	Tuberculosis (n = 37)	NTM (n = 8)	Cryptococcosis (n = 2)
Positive direct examination	9/28 (32)	11/23 (48)	4/5 (80)	0/2 (0)
Positive culture	21/28 (75)	15/23 (65)	2/4 (50)	0/2 (0)
Positive PCR	4/7 (57)	5/7 (71)	0/2 (0)	NA
Presence of yeasts or acid-fast bacilli on CHE	23/30 (77)	17/29 (59)	5/7 (71)	2/2 (100)

Abbreviations: CHE: Cytological and Histological Exam; NA: Not applicable; NTM: Non-tuberculous mycobacteria.

Epithelioid cells, granuloma and necrosis were found respectively in case of tuberculosis in 69% (22/32), 59% (19/32) and 78% (25/32), and in histoplasmosis in 45% (13/29), 28% (8/29) and 38% (11/29) (S1 Table).

Patients with inconclusive anatomical and/or cytopathological examination were analyzed (S2 Table). CHE was inconclusive in 19% of FNA and 28% of biopsies. In case of FNA, the low number of cells sampled (3/4) and the absence of microbiological staining performed (2/2) were the main causes. For biopsies, main limiting factors were the size of the sample, especially when only part of the lymph node was sent (24/28, 86%), and the absence of microbiological staining (9/28, 32%).

### Lymph nodes characteristics

Among available data, lymphadenopathy was of recent onset (< 3 months) in 68% of cases, even < 1 month in 40% of cases (Table 4). The most common biopsied sites were superficial (86%): cervical, inguinal and axillary in 44%, 25% and 17%, respectively. Lymph nodes were predominantly < 2 cm (86%), present on both sides of the diaphragm (69%). Lymph nodes > 5 cm were never associated with another lymph node of equivalent size, but could be associated with smaller ones. Associated radiological necrosis was described in 22% of cases. Clinical or radiological splenomegaly and hepatomegaly were present in 31% and 40% of cases, respectively.

**Table 4:** Clinical and radiological characteristics of the lymph nodes.

	No/Total (%) Median [IQR25%-75%]
**Time since onset of lymphadenopathy** (n = 47)	
< 1 month	19/47 (40)
[1-3 months]	13/47 (28)
[3 months-1 year]	10/47 (21)
> 1 year	5/47 (11)
**Biopsied site** (n = 132)	
Superficial sites	113/132 (86)
Cervical	58/132 (44)
Axillary	22/132 (17)
Inguinal	33/132 (25)
Deep sites	19/132 (14)
Spinal	2/132 (2)
Epitrochlear	1/132 (1)
Mediastinal	1/132 (1)
Para-Aortic	3/132 (2)
Mesenteric	12/132 (9)
**Size** (n = 145)	
< 2 cm	104/121 (86)
2-5 cm	51/116 (44)
> 5 cm	12/112 (11)
**Radiological necrosis**	23/103 (22)
**Consistency of lymph node (n = 55)**	
Soft	24/55 (44)
Firm/stonelike	22/55 (40)
Fluctuant	5/55 (9)
Fistulized	4/55 (7)
**Median number of lymph node area affected** ^*^	4 [2-6]
**Arrangement in relation to the diaphragm** (n = 128)	
One side	40/128 (31)
Both side	88/128 (69)
**Associated splenomegaly**	30/98 (31)
**Associated hepatomegaly**	42/105 (40)

*Clinical and/or radiological, bilateral involvement counting for 2 areas.

### Clinical and paraclinical associated data

Patients presented weight loss, fever and a WHO Performance Status > 2 in 67% (80/119), 45% (62/137) and 35% (46/130) of cases, respectively (Table 5). The other main clinical symptoms were cough (42/134, 31%), abdominal pain (34/135, 25%), and diarrhea (21/135, 16%).

**Table 5:** General clinical, radiological and biological data.

	No/Total (%)
**General state**	
Impaired WHO general performance status > 2 (PS > 2) ^*^	46/130 (35)
Weight loss	80/119 (67)
Fever (temperature > 38.2°C)	62/137 (45)
**Pulmonary abnormalities**	
Cough	42/134 (31)
Abnormal thoracic imaging^†^	86/106 (81)
Thoracic lymphadenopathy	55/106 (52)
Nodules	48/106 (45)
Condensations	37/106 (35)
Pleural effusion	18/106 (17)
**Digestive abnormalities**	
Abdominal pain	34/135 (25)
Diarrhea	21/135 (16)
**Laboratory results**	
Hemoglobin level < 12g/dL (normal values 13–18)	110/146 (75)
Neutrophil count < 1 500/mm3 (normal values 1500–7700)	36/146 (25)
Platelet count < 150 G/L (normal values 150–400)	29/146 (20)
C reactive protein > 50 mg/L (normal values < 5)	54/141 (38)
LDH level > 400 U/L (normal values 135–225)	23/117 (20)
Creatinine level > 100 µmol/L (normal values 60–100)	23/143 (16)
ASAT level > 34 U/L (normal values 10–34)	62/140 (44)
ALAT level > 55 U/L (normal values 10–55)	32/141 (23)
Alkaline phosphatase level > 150 U/L (normal values 40–130)	31/137 (23)
γ-Glutamyl transferase level > 50 U/L (normal values < 50)	75/139 (54)
ß-2-microglobulin level > 2.3 mg/L (normal values 1.2-2.2)	37/40 (93)
Positive galactomannan	2/16 (13)
Positive histoplasmosis serology	5/44 (11)
Positive ß-D-glucan	12/31 (39)
Positive TB-IGRA	22/49 (45)

Abbreviations: ALAT: ALanine AminoTransferase; ASAT: ASpartate AminoTransferase; LDH: Lactate DesHydrogenase; TB-IGRA: Tuberculosis Interferon Gamma Release Assay; WHO: World Health Organization.

*PS > 2: confined to bed or a chair more than 50% of waking hours;

†Chest X-ray and chest CT-scan.

Chest imaging was abnormal in 81% and revealed: thoracic lymphadenopathy (52%), parenchymal nodules (45%) and condensations (35%). Abdominal imaging found no relevant information compared to the clinical data. Anemia (hemoglobin level < 12g/dL), neutropenia (neutrophil counts < 1 500/mm^3^) and thrombocytopenia (platelets level < 150 G/L) were present in 75%, 25% and 20% of cases respectively. C-reactive protein was > 50mg/L in 38% of cases.

### Factors associated with AIDS-related opportunistic conditions

**Fig 2:**
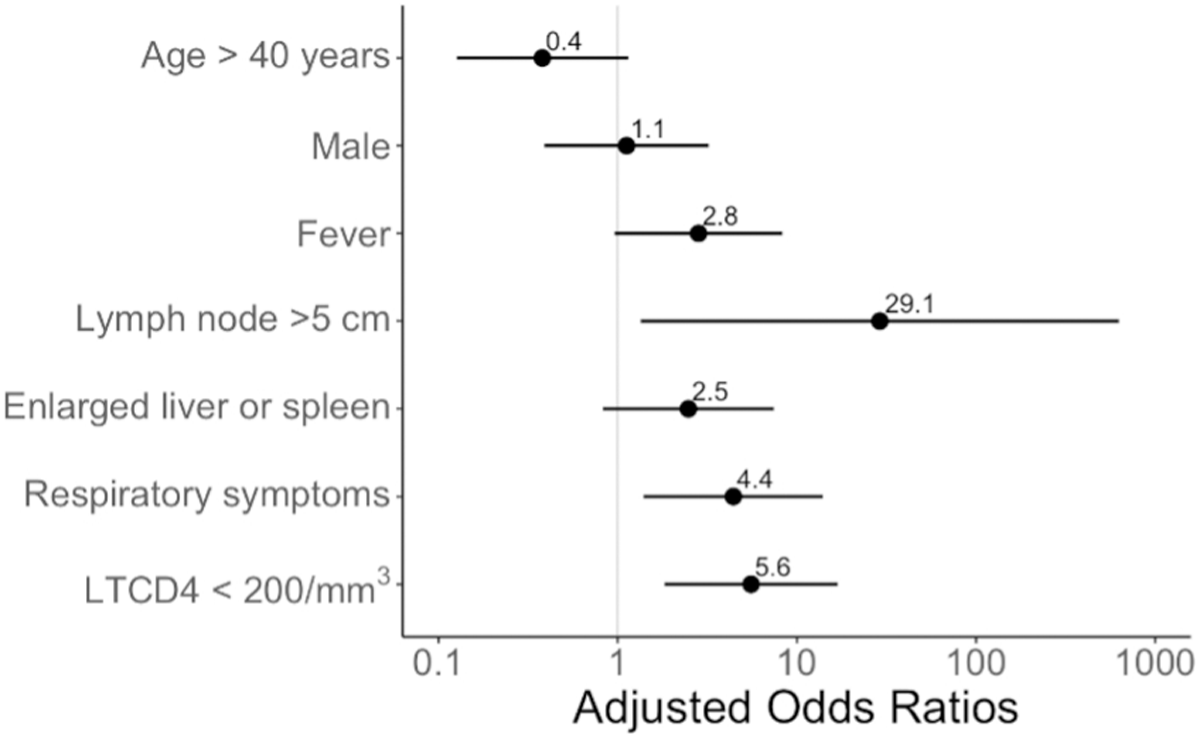
Forest plot synthetizing the multivariate analysis of main abnormalities orienting to an opportunistic cause.

**Fig 3:**
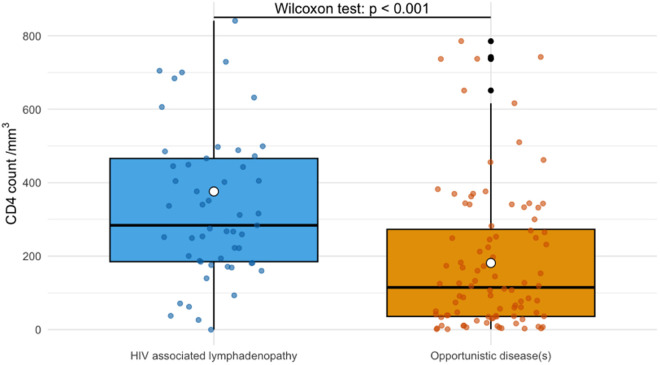
Boxplot of CD4 count according to whether the patient presented an opportunistic disease or not.

Ninety-five patients (62%) presented an AIDS-defining opportunistic condition, 42 (28%) an HIV-reactive or normal lymph node, and 15 (10%) patients were excluded from the analysis (metastasis and reactive or normal lymph node with extra-ganglionic opportunistic disease) (Table 6). Main symptoms associated with opportunistic conditions were lymphadenopathy > 5 cm, fever, pulmonary symptoms, hepatomegaly and diarrhea (Table 6 and Fig 2). Main paraclinical abnormalities were necrosis on medical imaging, low CD4 count (Table 6 and Fig 3), lower hemoglobin level, higher Aspartate AminoTransferase (ASAT), γ-Glutamyl transferase and C-reactive protein levels. Distribution on both sides of the diaphragm, presence of visceral or mediastinal lymphadenopathy, or pulmonary nodules did not differ between both groups.

**Table 6:** Main abnormalities between normal or HIV-reactive lymphadenitis and opportunistic diseases.

	Normal or HIV-reactive lymphadenitis	Opportunistic diseases^*^	p
**n**	42	95	
**Diagnosis made by lymph node biopsy** (and not FNA)	39/42 (92.9)	88/95 (92.6)	1.000
**Epidemiological data**			
Male gender (%)	19/42 (45.2)	67/95 (70.5)	**0.009**
Median age [IQR]	47 [37, 54]	41 [34, 49]	**0.044**
Living in Cayenne and surroundings (pseudo urban) (%)	25/36 (69.4)	51/89 (57.3)	0.291
Regular declared profession (%)	6/31 (19.4)	21/72 (29.2)	0.427
Heterosexual transmission of HIV (%)	30/40 (75.0)	85/94 (90.4)	**0.038**
History of opportunistic (AIDS ranking) disease (%)	11/41 (26.8)	26/94 (27.7)	1.000
HTLV (%)			0.641
Positive serology (%)	2/38 (5.3)	5/80 (6.2)	
Positive PCR (%)	1/38 (2.6)	1/80 (1.2)	
**Lymph node characteristics**			
Clinical superficial lymphadenopathy (%)	32/39 (82.1)	87/92 (94.6)	0.052
> 5 cm (%)	0/37 (**0.0**)	10/80 (12.5)	0.058
[2–5] cm (%)	15/37 (40.5)	37/85 (43.5)	0.914
[1–2] cm (%)	30/39 (76.9)	66/85 (77.6)	1.000
Cervical area (%)	20/20 (100.0)	67/67 (100.0)	NA
Axillary area (%)	20/20 (100.0)	41/41 (100.0)	NA
Inguinal area (%)	22/22 (100.0)	45/46 (97.8)	1.000
Firm consistency (%)	2/11 (18.2)	15/32 (46.9)	0.186
Deep sites (visceral) (%)	13/25 (52.0)	50/70 (71.4)	0.129
Mediastinal (%)	7/25 (28.0)	36/70 (51.4)	0.074
Abdominal (%)	10/25 (40.0)	40/70 (57.1)	0.215
Necrosis (%)	1/25 (4.0)	20/67 (29.9)	**0.019**
Distribution on both sides of the diaphragm (%)	22/37 (59.5)	62/92 (67.4)	0.515
Hepatomegaly (%)	7/31 (22.6)	40/81 (49.4)	**0.018**
Splenomegaly (%)	7/31 (22.6)	35/81 (43.2)	0.072
**Clinical and radiographical presentation**			
Performans Status > 2 (%)	3/35 (8.6)	16/83 (19.3)	0.242
Weight loss (%)	14/32 (43.8)	57/76 (75.0)	**0.004**
Fever (> 38.2°C) (%)	7/35 (18.9)	50/86 (58.1)	**< 0.001**
Respiratory signs and symptoms (%)	10/36 (27.8)	48/87 (55.2)	**0.010**
Cough (%)	6/36 (16.7)	33/87 (37.9)	**0.036**
Thoracic nodules (chest X-ray and/or CT-scan) (%)	8/25 (32.0)	32/76 (42.1)	0.509
Digestive signs and symptoms (%)	12/37 (32.4)	42/87 (48.3)	0.153
Diarrhea (%)	2/37 (5.4)	19/86 (22.1)	**0.046**
Neurological signs and symptoms (%)	10/37 (27.0)	19/86 (22.1)	0.719
Cutaneous signs^†^ (%)	4/36 (11.1)	25/86 (29.1)	0.058
Oral mucosal lesions^‡^ (%)	1/36 (2.8)	23/86 (26.7)	**0.005**
**Laboratory results**			
Median CD4 cell count (/mm3) [IQR]	327 [217, 468]	115 [36, 273]	**< 0.001**
CD4 cell count < 200/mm3 (%)	9/40 (22.5)	60/92 (65.2)	**< 0.001**
Median CD8 cell count (/mm3) [IQR]	952 [636, 1284]	588 [210, 956]	**0.001**
Median viral load (log10 copies/ml) [IQR]	3.6 [1.7, 4.8]	4.9 [2.9, 5.7]	**0.002**
Median hemoglobin level (g/dl) [IQR]	11.8 [10.3, 13.0]	10.1 [8.6, 11.5]	**0.001**
Median platelets count (G/L) [IQR]	246 [196, 301]	213 [170, 310]	0.494
Median neutrophils count (/mm3) [IQR]	2310 [1380, 3290]	2760 [1480, 4570]	0.176
Median albumin level (g/l) [IQR]	32.6 [29.3, 36.1]	28.0 [21.9, 33.5]	**0.011**
Median ASAT level (UI/L) [IQR]	24 [21, 30]	39 [25, 58]	**0.001**
Median ALAT level (UI/L) [IQR]	18 [12, 28]	23 [16, 37]	**0.048**
Median γ-Glutamyl transferase level (UI/L) [IQR]	33 [22, 78]	74 [37, 134]	**0.002**
Median Alkaline phosphatase level (UI/L) [IQR]	85 [65, 125]	110 [75, 162]	0.068
Median C reactive protein level (mg/L) [IQR]	10 [4, 42]	39 [11, 91]	**0.005**
Median LDH level (UI/L) [IQR]	230 [209, 301]	281 [225, 395]	0.076

Abbreviations: ALAT: ALanine AminoTransferase; ASAT: ASpartate AminoTransferase; FNA: Fine Needle Aspiration; HTLV: Human T-Lymphotropic Virus; IQR: interquartile ranges; LDH: Lactate DesHydrogenase; PCR: Polymerase Chain Reaction.

*Histoplasmosis, tuberculosis and other mycobacterial infections, cryptococcosis, Kaposi sarcoma, lymphoproliferative disorder;

†Mainly composed of prurigo and herpetic lesions;

‡Mainly composed of oral candidiasis.

There did not appear to be differences in terms of positive indirect tests (tuberculosis Interferon Gamma Release Assay, Histoplasma serology, galactomannan antigen, ß-D-glucan, ß-2-microglobulin) between the HIV-reactive, tuberculosis, histoplasmosis, lymphoma or metastasis groups (S3 Table). Tuberculosis was the only cause associated with fluctuant or fistulized lymphadenopathy (2 patients each) and the main cause of radiological necrosis.

## Discussion

We found that AIDS-defining opportunistic infections were responsible for 53% of lymphadenopathy, when lymphoproliferative disorders were responsible for 11% of cases. In the Amazon area, a similar study conducted among 120 patients in Colombia, found tuberculosis (48%) and reactive lymphadenitis (33%) as the main etiologies of lymph nodes enlargement [13]. In contrast, histoplasmosis was only found in 13% of cases, with a rural-urban gradient (28.5% vs 9.1%). Similar results were also found in 210 South-Brazilian patients, with only 2% of histoplasmosis versus 50% of mycobacteria and 23% of reactive lymphadenitis [14]. Patients included in this study also seem to mainly come from urban area. In those studies, however, it seems that no mycological exam was performed and special staining in CHE was not systematic either. The estimated annual incidence of histoplasmosis cases in PLHIV is similar in French Guiana and Colombia but lower in Brazil [15], with a highest endemicity in the North-East regions of Brazil [16]. Our more elevated prevalence of lymphadenopathy due to histoplasmosis could be explained by our more systematic review of histoplasmosis in French Guiana.

We found a low number of AIDS-associated Kaposi sarcoma, when it is described as a classical cause of lymphadenopathy in an autopsy series [17], probably due to the fact that the diagnosis of Kaposi’s disease was made via skin lesion biopsies, easier to perform, and did not require lymph node biopsy. For the same reason, HHV8 serology or PCR was rarely performed because diagnosis was mainly made by clinical examination.

Almost half of patients presented with lung nodules, without significant difference between patients with benign or opportunistic diseases. Their presence, as much as positive indirect markers such as tuberculosis Interferon Gamma Release Assay, *Histoplasma* serology, galactomannan antigen, ß-D-glucan seem to be of limited use to guide the diagnosis, in a population exposed to tuberculosis and histoplasmosis and at risk of asymptomatic forms, however it is difficult to conclude due to the numerous missing data. Positive ß-2-microglobulin, classically described as a marker of tumor burden in LPD [18,19], has also been described as a marker of HIV’s activity itself and CD4 count [20,21]. In the population of the present study, it seems to reflect the global level of immunosuppression, as much as the presence of replicative pro-oncogenic viruses and does not suggest a neoplastic cause.

Granuloma with caseous necrosis and multinuclear giant cell, generally described in tuberculosis [22], can also be found in histoplasmosis [23]. Our results emphasize the necessity of multiplying samples gathered, since pathology seems to yield greater numbers of positive results than microbiological direct examination and can also produce faster results than fungal or mycobacterial culture, but requires a trained, epidemiologically aware examiner [24].

Main factors associated with an opportunistic cause were fever, weight loss, presence of a lymph node > 5 cm and CD4 cell count < 200/mm3. In the literature, larger lymph node size was already described as a predictive factor for non-reactive causes [2], malignant lymphoma [25] or infectious disease [26]. Weight loss [2,13], fever [2] and CD4 cell count < 50/mm3 [13] were also described as less predictive of reactive lymphadenitis.

This study contains selection biases since patients were selected based on pathology’s samples rather than clinical presentation. Hence, lymph nodes of lower sizes without general signs were less likely to be addressed and sampled. Patients were not comparable over the inclusion period since before 2016, no systematic antiretroviral treatment was recommended in our territory in asymptomatic patients with CD4 cell counts > 500/mm^3^ [10,11]. Despite its biases, this study is exhaustive over 17 years, and relatively comprehensive, as all lymph nodes samples were sent to Cayenne hospital for pathology.

While analyzing the etiologies of lymphadenopathy in PLHIV, tuberculosis endemicity in the region studied should be a major factor to consider. In countries where it is highly endemic such as India [27], China [28], or South Africa [29], it represents 30–60% of causes of lymphadenopathy, while in countries of low endemicity such as the USA, the main etiologies are reactive adenitis (49.5%) and malignancies (42.9%) [2]. The present study suggests that in regions where histoplasmosis is endemic, the clinical reasoning behind diagnostic assessments should be the same as for tuberculosis.

In conclusion, in French Guiana, the main etiologies of lymphadenopathy in PLHIV were opportunistic infections with histoplasmosis and tuberculosis being the main etiologies over HIV-reactive lymphadenitis. Histoplasmosis, already known as the first AIDS-defining condition in our territory was also the first etiology of lymphadenopathy in PLHIV.

We do not believe that the presence of lung nodules or positive indirect markers for tuberculosis or histoplasmosis is relevant to guide diagnosis in immunosuppressed patients in the Amazons, where both infections are endemic with high risk of former asymptomatic exposure. In the presence of lymphadenopathy in these patients, low CD4 count and lymph nodes > 5 cm should quickly lead to lymph node biopsy in search for associated opportunistic infections or lymphoproliferative disorders.

## Supporting information

S1 Table Anatomical and cytological characteristics of fine needle aspirations and biopsies (after exclusion of patients presenting several causes of lymphadenopathy and/or extra lymphatic infection). (DOCX)

S2 Table Patients with inconclusive anatomical and/or cytopathological examination. (DOCX)

S3 Table Comparative data between the 5 main etiological groups (after exclusion of patients with multiple diagnoses). (DOCX)
