# Supplementary material for: Etiological spectrum and diagnostic features of lymphadenopathy in People Living with HIV in French Guiana: A 17-years multicenter retrospective case series
Source: PLoS Negl Trop Dis. 2025 Sep 22;19(9):e0013558. doi: 10.1371/journal.pntd.0013558 (PMC12478962; doi:10.1371/journal.pntd.0013558)
Supplement: S1 Table — (DOCX) [file pntd.0013558.s001.docx]

**S1_Table. Anatomical and cytological characteristics of lymph node fine needle aspirations and biopsies** (after exclusion of patients presenting several causes of lymphadenopathy and/or extra lymphatic infection) (No/Total (%))

|  | Reactive to HIV (n = 30) | Tuberculosis  (n = 32) | Histoplasmosis  (n = 29) | Lymphoma  (n = 12) | Metastasis  (n = 9) |
| --- | --- | --- | --- | --- | --- |
| **Diagnosis** |  |  |  |  |  |
| Positive CHE | 30/30 (100) | 18/32 (56) | 21/29 (72) | 11/12* (92) | 9/9 (100) |
| **Anatomical abnormalities** |  |  |  |  |  |
| Epithelioid cells | 1/27 (4) | 22/32 (69) | 13/29 (45) | 0/12 (0) | 0/8 (0) |
| Granuloma | 1/27 (4) | 19/32 (59) | 8/29 (28) | 2/12 (17) | 0/8 (0) |
| Necrosis | 0/28 (0) | 25/32 (78) | 11/29 (38) | 2/12 (17) | 0/9 (0) |
| Acid-fast bacilli | 0/20 (0) | 16/29 (55) | 0/19 (0) | 0/8 (0) | 0/9 (0) |
| Yeasts | 0/21 (0) | 1/23 (4) | 21/25 (84) | 0/9 (0) | 0/9 (0) |

Abbreviations: CHE: Cytological and Histological Exam; NA: Not Applicable; PCR: Polymerase Chain Reaction.

* diagnosis made on another organ.
