# Supplementary material for: Etiological spectrum and diagnostic features of lymphadenopathy in People Living with HIV in French Guiana: A 17-years multicenter retrospective case series
Source: PLoS Negl Trop Dis. 2025 Sep 22;19(9):e0013558. doi: 10.1371/journal.pntd.0013558 (PMC12478962; doi:10.1371/journal.pntd.0013558)
Supplement: S2 Table — (DOCX) [file pntd.0013558.s002.docx]

**S2_Table. Patients with inconclusive anatomical and/or cytopathological examination**

|  | **No/Total (%)** |
| --- | --- |
| **Type of examination performed** |  |
| CHE in FNA | 21/24 (88) |
| Inconclusive CHE in FNA | 4/21 (19) |
| Not enough cells in sample | 3/4 (75) |
| Microbiological coloration not made* | 2/2 (100) |
| Diagnosis made by lymph node’s biopsy CHE | 3/4 (75) |
| Diagnosis made by CHE in another organ | 1/4 (25) |
| CHE in Biopsy | 100/142 (70) |
| Inconclusive CHE in biopsy | 28/100 (28) |
| Low size of sample or fragments and not whole adenopathy | 24/28 (86) |
| Major necrosis | 3/28 (11) |
| Microbiological coloration not made* | 9/28 (32) |
| Diagnosis made by microbiology^†^ | 28/28 (100) |

Abbreviations: CHE: Cytological and Histological Exam; FNA: Fine Needle Aspiration.

*in case of infectious disease; ^†^ necrosis, non-specific adenitis and epithelioid and giant cell granuloma were considered as inconclusive diagnosis as they could not be assigned to an etiological group.
