# Supplementary material for: Etiological spectrum and diagnostic features of lymphadenopathy in People Living with HIV in French Guiana: A 17-years multicenter retrospective case series
Source: PLoS Negl Trop Dis. 2025 Sep 22;19(9):e0013558. doi: 10.1371/journal.pntd.0013558 (PMC12478962; doi:10.1371/journal.pntd.0013558)
Supplement: S3 Table — (DOCX) [file pntd.0013558.s003.docx]

**S3_Table. Comparative data between the 5 main etiological groups** (after exclusion of patients with multiple diagnoses) (No/Total (%))

|  | Reactive to HIV (n = 30) | Tuberculosis  (n = 32) | Histoplasmosis  (n = 29) | Lymphoma  (n = 12) | Metastasis (n = 9) |
| --- | --- | --- | --- | --- | --- |
| **Sex ratio M/F** | 1 | 2.7 | 1.4 | 4.5 | 1.25 |
| **Age < 40 years** | 10/30 (33) | 16/26 (62) | 10/24 (42) | 0/11 (0) | 0/9 (0) |
| **Consumption of toxic substances** | 7/24 (29) | 13/21 (62) | 10/18 (56) | 4/8 (50) | 3/7 (43) |
| Alcohol | 5/24 (21) | 11/21 (52) | 7/18 (39) | 2/8 (25) | 3/7 (43) |
| Tobacco | 4/24 (17) | 6/21 (29) | 5/18 (28) | 2/8 (25) | 1/7 (14) |
| Marijuana | 1/24 (4) | 2/21 (10) | 3/18 (17) | 2/8 (25) | 1/7 (14) |
| Crack | 1/24 (4) | 3/21 (14) | 3/18 (17) | 2/8 (25) | 0/7 (0) |
| **ART at diagnosis*** | 9/29 (31) | 6/24 (25) | 6/24 (25) | 6/10 (60) | 9/9 (100) |
| **CD4 < 200/mm3** | 5/30 (17) | 17/26 (65) | 14/24 (58) | 4/8 (50) | 1/7 (14) |
| **VL < 40 cp/ml*** | 1/29 (3) | 4/23 (17) | 3/22 (14) | 1/8 (13) | 4/8 (50) |
| **Pro-oncogenic virus**^†^ |  |  |  |  |  |
| HCV | 1/29 (3) | 0/26 (0) | 0/23 (0) | 1/10 (10) | 0/9 (0) |
| HBV | 1/29 (3) | 3/25 (12) | 1/23 (4) | 2/10 (20) | 0/9 (0) |
| CMV | 1/29 (3) | 4/24 (17) | 4/21 (19) | 1/10 (10) | 0/6 (0) |
| EBV | 2/13 (15) | 2/7 (29) | 1/7 (14) | 4/7 (57) | 0/0 (0) |
| HHV8 | 0/6 (0) | 0/4 (0) | 0/3 (0) | 1/4 (25) | 0/0 (0) |
| HTLV | 0/27 (0) | 0/22 (0) | 0/21 (0) | 0/8 (0) | 0/9 (0) |
| HPV | 5/6 (83) | 3/3 (100) | 0/1 (0) | 0/0 (0) | 0/1 (0) |
| **Consistency and radiological characteristics** |  |  |  |  |  |
| Fluctuant | 0/8 (0) | **2/11 (18)** | 0/4 (0) | 0/5 (0) | 0/2 (0) |
| Fistulized | 0/8 (0) | **2/11(18)** | 0/4 (0) | 0/5 (0) | 0/2 (0) |
| Radiological necrosis | 0/20 (0) | 6/18 (33) | 4/17 (24) | 1/9 (11) | 1/5 (20) |
| **Thoracic nodules (chest X-ray and/or CT-scan)** | 7/14 (50) | 8/16 (50) | 8/16 (50) | 3/6 (50) | 3/4 (75) |
| **Laboratory results** |  |  |  |  |  |
| ß-2-microglobulin > 2.3 mg/L | 7/8 (88) | 3/4 (75) | 6/6 (100) | 4/5 (80) | 0/0 (0) |
| Positive galactomannan | 0/1 (0) | 0/3 (0) | 2/16 (13) | 2/9 (22) | 0/0 (0) |
| Positive histoplasmosis serology | 1/7 (14) | 0/5 (0) | 11/19 (58) | 2/9 (22) | 0/7 (0) |
| Positive ß-D-glucan | 3/6 (50) | 2/6 (33) | 2/4 (50) | 1/8 (13) | 0/1 (0) |
| Positive TB-IGRA | 6/10 (60) | 9/9 (100) | 9/11 (82) | 2/6 (33) | 0/1 (0) |

Abbreviations: ART: Anti Retoviral Therapy; CMV: Cytomegalovirus; EBV: Epstein–Barr virus; HBV: Hepatitis B virus; HCV: Hepatitis C virus; HHV8: Human Herpes Virus 8; HPV: Human Papilloma Virus; HTLV: Human T-Lymphotropic Virus; TB-IGRA: Tuberculosis Interferon Gamma Release Assay; VL: Viral Load.

* patients with a newly diagnosed HIV infection and patients included before 2016 and presenting more than 500 CD4/mm3 were not included since it was not strictly recommended to treat them that time following national recommendations; ^†^ positive PCR.
